# Clinical Effectiveness of a Novel Caffeine Nano-Cream for Cellulite Reduction: A Randomised Double-Blind Trial

**DOI:** 10.3390/pharmaceutics18020151

**Published:** 2026-01-24

**Authors:** Thellie Ponto, Christofori M. R. R. Nastiti, Giuseppe Luna, Vânia R. Leite-Silva, Brioni R. Moore, Anthony Wright, Heather A. E. Benson

**Affiliations:** 1 Curtin Medical School, Curtin University, Perth, WA 6102, Australiabrioni.moore@curtin.edu.au (B.R.M.); 2 Division of Pharmaceutics and Pharmaceutical Technology, Faculty of Pharmacy, Sanata Dharma University, Yogyakarta 55282, Indonesia; 3 Departamento de Ciências Farmacêuticas, Instituto de Ciências Ambientais, Químicas e Farmacêuticas, Universidade Federal de São Paulo, Rua São Nicolau, 210, Diadema 09913-030, Brazil; 4 Curtin Medical Research Institute, Curtin University, Perth, WA 6102, Australia; 5 School of Allied Health, Curtin University, Perth, WA 6102, Australia

**Keywords:** caffeine, chemical penetration enhancers, nano-cream formulations, skin targeting, clinical trial, cellulite, topical delivery, cosmeceutical

## Abstract

**Background**: Caffeine (CAF), whether extracted from plants or synthesised as a chemical compound, is considered the safest among other xanthine alkaloids. Novel nano-cream formulations have been successfully developed and evaluated to increase the potential of caffeine as a skin cosmeceutical, targeting the minimisation of cellulite appearance. **Methods**: Nano-cream formulations were prepared through a process of hot-temperature emulsification, in a variety of homogeniser combinations. **Results**: When chemical penetration enhancers (CPEs) (lanolin, transcutol, and propylene glycol), either alone or in combination, were incorporated into the nano-cream formulations, the permeation of CAF through skin increased. All nano-cream formulations achieved sustained delivery of CAF into and through the skin over 8 h (IVPT). Quantification of CAF from skin tissues was achieved using high-performance liquid chromatography (HPLC). The nano-cream formulation containing lanolin (LAN) showed the highest CAF permeation (8.829 ± 1.472 µg/cm^2^/h) through the skin compared to CAF in an aqueous solution (2.533 ± 0.480 µg/cm^2^/h) and a commercial CAF cellulite product with the same CAF concentration (2.827 ± 0.555 µg/cm^2^/h). Therefore, 2% CAF nano-cream formulation containing LAN was chosen for clinical testing. A double-blind, randomised, placebo-controlled paired trial was conducted, in which each volunteer applied active and placebo creams to the upper thighs twice daily for 12 weeks. The effect of the cream on skin appearance was monitored over 12 weeks. The primary outcome measures were reduced cellulite scores from 3.96 (95% CI: 3.16–4.76) to 2.50 (95% CI: 1.70–3.30) (active) compared with placebo from 3.88 (95% CI: 3.08–4.67) to 2.83 (95% CI: 2.03–3.63). The effect sizes (E.S.) indicated a moderate effect for the active CAF nano-cream formulation (E.S. = 0.475), while the placebo (E.S. = 0.286) had a small effect. **Conclusion**: We concluded that our optimised 2% CAF nano-cream formulation containing LAN offered an effective formulation strategy for enhancing skin penetration in the IVPT study. The LAN nano-cream formulation demonstrated efficacy and tolerability, both objectively and subjectively, in a human clinical trial.

## 1. Introduction

Cellulite (ginoide hydrolipodystrophy) is prevalent in women, affecting 80–90% of postpubertal females [1,2,3,4]. It is a metabolic condition that affects the dermo-epidermal junction, characterised by increased amounts and sizes of fat deposits known as adipocytes [5]. It manifests predominantly on the buttocks and thighs [1,6,7]. Adipose tissue protrudes into the dermis, resulting in a skin texture appearance commonly described as “orange peel” or “cottage cheese-like” [8]. Treatment approaches to reduce the visibility of cellulite include surgical interventions, lipolysis techniques such as cryolipolysis, and non-invasive approaches like laser therapy, radiofrequency, and acoustic wave therapies. These procedures require a visit to a specialised clinic, are expensive [9,10], and present complications related to pain [1,11], erythema [1,12], bruising [1,11,13,14,15], and thermal skin damage due to exposure to excessive heat or cold [16]. This study evaluates the efficacy of a self-administered topical treatment for cellulite that can be applied at a low cost and exhibits minimal side effects.

Various active ingredients, including methylxanthine alkaloids such as caffeine (CAF), are utilised as natural topical treatments for cellulite to regulate fat storage in adipocyte tissue [1,17,18,19,20,21,22]. CAF, at concentrations of 1% to 2% [17,23], has been proven effective in reducing cellulite through numerous clinical trials [1,24,25], although some marketed topical anti-cellulite products have higher concentration ranges from 3% to 7% CAF [6,26,27]. For example, Lupi et al. [28] evaluated the effectiveness of an anti-cellulite product containing 7% of CAF solution (Elancyl^®^ Chrono Active). After one month of treatment, a significant reduction in thigh circumference was observed in more than 80% of treated participants, with a 67.7% decrease in hip circumference. Topical CAF specifically targets fat tissue cells [23], enhancing lipolysis (breakdown of triglycerides stored in fat cells in the skin) [6,20,23], stimulating peripheral microcirculatory flow [3], and reducing oedema (inflammation) [17,29]. CAF acts by inhibiting phosphodiesterase (PDE) activity, enhancing cyclic adenosine monophosphate (cAMP) activity in adipocytes [3,6,19,30], and activating hormone-sensitive lipase (HSL) [20]. Decreasing cAMP levels inhibits lipolysis, while increasing cAMP stimulates protein kinase A, which regulates HSL. The conversion of cAMP to noncyclic 5′-AMP reflects PDE activity. Activation of HSL results in fat breakdown during lipolysis and also improves blood circulation [23]. CAF also supports lymphatic drainage by eliminating excess fat and toxins during the lipolysis process.

We developed semi-solid nano-cream formulations containing CAF to enhance skin permeation [31]. We characterised the physical properties and ex vivo skin permeation of CAF using Franz diffusion cells to optimise the formulation. The strongest candidate formulation was then evaluated in a clinical study to assess product efficacy and safety in human participants [31].

## 2. Materials and Methods

### 2.1. Materials

CAF of analytical grade (CAS# 58-08-2 (C0750) (anhydrous, with 99% purity level), isopropyl myristate (IPM) (CAS# 110-27-0), and butylated hydroxytoluene (BHT) (CAS# B-1378) were purchased from Sigma-Aldrich (North Ryde, NSW, Australia). Cetearyl alcohol (stearyl alcohol and cetyl alcohol) (stearyl alcohol: CAS# 112-92-5; cetyl alcohol: CAS# 36653-82-4), ceteareth-20 (CAS# 68439-49-6), and dimethicone 350 CST (CAS# 9006-65-9) were purchased from New Directions Laboratory (Marrickville, NSW, Australia). Lanolin USP anhydrous (CAS# 8006-54-0) was purchased from PCCA (Matraville, NSW, Australia). Extra virgin olive oil (CAS# 8001-25-0) and optiphen^TM^ plus (phenoxyethanol, caprylyl glycol and sorbic acid) (phenoxyethanol: CAS# 122-99-6; caprylyl glycol: CAS# 1117-86-8; sorbic acid: CAS# 110-44-1) were purchased from Range Products (Welshpool, WA, Australia). DL-alpha-tocopheryl acetate (CAS# 7695-91-2) was purchased from Australian Wholesale Oils (Rydalmere, NSW, Australia). Triethanolamine (TEA) (CAS# 102-71-6) and ethylene diamine tetra-acetic acid (EDTA) (CAS# 139-33-3) were purchased from BDH Laboratory Supplies (Poole, Dorset, UK), distributed by Merck Pty Ltd. (Kilsyth, VIC, Australia). Carbopol^®^ 940 (CAS# 9003-01-4) was a gift from Lubrizol International Inc. (Silverwater, NSW, Australia). Propylene glycol BP (CAS# 57-55-6) was purchased from PharmAust Manufacturing (Malaga, WA, Australia). Transcutol^®^ P (diethylene glycol monoethyl ether-CAS# 111-90-0) was a gift from Gattefossé (Saint-Priest, France). Veet™ hair removal cream (containing potassium thioglycolate) was purchased from Reckitt Benckiser Pty Ltd. (Sydney, NSW, Australia). Methanol HPLC grade and hexane were from Fisher Chemical (Pittsburgh, PA, USA). Deionised water was passed through a Milli-Q RC apparatus (Millipore Corporation, Bedford, MA, USA). PBS (pH 7.4) (phosphate-buffered saline solution) was prepared by dissolving 8.0 g NaCl, 0.2 g KCl, 1.44 g Na_2_HPO_4_, and 0.24 g KH_2_PO_4_ into 1 L deionised water and adjusting the pH 7.4 with 0.1 N potassium hydroxide. Potassium chloride and sodium chloride were purchased from Chem-Supply Australia Pty Ltd. (Gillman, SA, Australia). Potassium dihydrogen orthophosphate was from Thermo Fisher Scientific (Scoresby, VIC, Australia). Di-sodium hydrogen orthophosphate anhydrous was purchased from Merck Pty Ltd. (Bayswater, VIC, Australia). Ultra cellulite cream (marketed) was purchased from Green Organics (LOT #CO91633) (Salt Lake City, UT, USA). The manufacturer’s specifications list key ingredients as follows: CAF 2%, stabilising agents, and emollients like cetearyl alcohol, polysorbate 60, and stearic acid. *Rhodophyceae* extract and vitamin B5, C, and E provide skin-enhancing properties. These ingredients are combined to create a cream formulation that includes xanthan gum, glycols, shea butter, avocado oil, and deionised water. The formulation also contains a preservative.

### 2.2. HPLC Instrumentation and Conditions

Chromatographic separation was performed using an Agilent™ 1200 system (Agilent Technologies, Waldbronn, Germany) using a Jupiter C18 5 µm column (150 mm × 4.6 mm) with a Guard Cartridge (C18, 4 mm × 3 mm), both from Phenomenex (Lane Cove, Australia), and isocratic flow of mobile phase (methanol/water ratio = 30:70) at 1 mL/min (chromatography adapted from Naegele [32], with some modifications). The autosampler was set to a controlled temperature of 10 °C.

### 2.3. Design of CAF Nano-Cream Formulations

The composition of eight nano-cream formulations containing CAF is documented in Table 1. The nano-creams were prepared using the method described by Wojciechowska et al. [33] with some modifications, involving a hot emulsification process and low-energy input. Briefly, both the aqueous and oil phases were heated separately until reaching 70 °C. In phase A, all oil-soluble compositions were melted at 70 °C, while all aqueous-soluble compositions (phase B), including 2% (*w*/*w*) CAF, were dissolved in water and heated until temperature reached 70 °C. Next, phase A was incorporated into phase B and blended using a homogeniser (DIAX 900, Heildoph Instruments GmbH & Co. KG, Schwabach, Germany) at 8000 rpm (speed no. 1) for 15 min to form an emulsion. The mixture was then stirred continuously until the temperature dropped to 50 °C. Optiphen plus^®^ (preservative) and DL-alpha-tocopheryl acetate were added at 8000 rpm for 15 min to achieve a uniform consistency. Finally, the drop-wise addition of TEA was adjusted to achieve a pH below 6, which is suitable for skin application [34] (MColorpHast, Darmstadt, Germany). The pH of the cream was further measured accurately using a calibrated benchtop pH metre (HI 8519 N; Hanna Instruments, Woonsocket, RI, USA). The emulsion was allowed to cool to ambient temperature.

### 2.4. Physical Evaluation of Topical Creams

As detailed below, the CAF creams were physically characterised in appearance (visually organoleptic) and pH in cream and droplet size.

#### 2.4.1. Organoleptic Characteristics

After preparation, all CAF nano-cream formulations were tested for appearance, colour, consistency, homogeneity, phase separation, and texture at room temperature. Homogeneity and texture were assessed by pressing a small amount of the formulated cream between the thumb and index finger. The consistency and presence of coarse particles were used to evaluate the homogeneity and texture of the formulations. These characteristics were observed visually, which is considered adequate for aesthetic perception [35,36,37].

#### 2.4.2. pH Measurement

The pH of each cream was tested immediately after preparation using pH strips and the accompanying colour chart at 5 to 6 (McolorpHast, Darmstadt, Germany). The pH of the cream was further determined accurately using a calibrated benchtop pH metre (HI 8519 N; Hanna Instruments, Woonsocket, RI, USA). After cooling, the creams were separated by high-speed centrifugation (model: Optima XE-100; rotor type: 70.1 Ti; Beckman Coulter Inc., Brea, CA, USA) at 55,000 rpm for 4 h and then the separated aqueous phase was measured with a calibrated pH metre.

#### 2.4.3. Droplet Size

The droplet size distribution of the CAF-loaded nano-cream formulation was determined by photon correlation spectroscopy (PCS) using a Zetasizer Nano™ ZSP (Malvern Panalytical, Malvern, Worcestershire, UK) following 100 times dilution with deionised water to prevent multiple scattering [38].

### 2.5. In Vitro Skin Penetration/Permeation Study (IVPT)

Piglet skin, which is similar in anatomy and physiology to human skin, serves as a suitable model and appropriate surrogate due to the limited supply of human skin [39,40,41]. Full-thickness skin was obtained from newborn Yorkshire piglets that died within 24 h, collected from a local veterinarian. The skin was peeled from the body and a surgical scalpel was used to remove excessive fat tissues. Veet^®^ depilatory cream was used for 10 min to facilitate hair removal. This method has been previously validated on piglet skin and shown to cause no skin damage [39]. The skin surface was rinsed thoroughly with PBS (pH 7.4) to remove dirt and cream, then blotted dry before being stored at −20 °C until use.

#### 2.5.1. Experimental Set-Up

Three piglet skin donors were used for each experiment, providing 6–9 replications. Full-thickness excised skin was thawed at room temperature prior to the experiment. Skin thickness (400–600 µm) was measured by a digital vernier calliper (Model K11100, Kincrome Australia Pty Ltd., Scoresby, Australia). The skin was cut into suitable-sized circles and mounted in the Franz diffusion cells (stratum corneum; SC side up), and the barrier integrity was checked by measuring electrical resistance with a digital multimeter (UNI-T^®^, UT58 series, Opava-Předměstí, Czech Republic) with PBS (pH 7.4) in the donor and receptor compartments allowed to equilibrate in the water bath at 35 °C for 20 min. One probe of the multimeter was inserted via the arm into receptor fluid and the other was applied to the donor solution. PBS (pH 7.4) in the donor compartment was then removed and the skin surface was wiped dry (Kimtech Science Kimwipes^®^, Kimberly-Clark Professional, Milsons Point, Australia). The receptor compartment was filled with a mixture of PBS (pH 7.4) and methanol (50:50). A magnetic stirrer was added to the receptor compartment, stirred at 600 rpm/min and maintained in a water bath system at 35 °C (skin surface temperature 32 °C) [42]. Next, 0.2 g of each nano-cream formulation, the marketed CAF product, or CAF in aqueous solution (control) was applied to the skin surface and the donor compartment covered with Parafilm M^®^ Laboratory Film (Greenwich, CT, USA). Samples (total receptor fluid volume) were withdrawn from the receptor compartment at six time points (0.5, 1, 2, 4, 6, and 8 h) for HPLC analysis and immediately replaced by fresh pre-warmed receptor solution (total replacement).

#### 2.5.2. Skin Distribution Study

After the 8 h sampling was completed, the amount of CAF in the SC was assessed using a tape stripping process. The remaining cream in the donor compartment was removed and the skin was washed to remove any remaining formulation (washing fluid retained for a mass balance calculation). The skin surface was gently dried with Kimwipes^®^ and treated to separate the SC from the remaining skin tissue (E + D + F = epidermis/dermis/follicle) by application of D-Squame^®^ adhesive tapes (diameter of 22 mm; CuDerm, Dallas, TX, USA) using a D-Squame disc^®^ applicator [43]. Ten tape strips were used; the first two strips were assumed to include unabsorbed CAF (kept aside for mass balance study). The skin was then sectioned and weighed prior to CAF extraction. CAF in tapes and sectioned skin were soaked in a PBS (pH 7.4):MeOH (50:50) mixture and stirred magnetically at 600 rpm/min for 2 h at room temperature. The supernatant fluid was collected and centrifuged at 15,000 rcf for 10 min (25 °C) to precipitate fatty tissue and the CAF content was then analysed by HPLC.

### 2.6. Data Analysis

For the IVPT, the cumulative amount per area (µg/cm^2^) versus time (h) was plotted and used to determine the steady-state flux (*Jss*; μg/cm^2^/h), permeability coefficient (Kp; cm/h), lag time, and enhancement ratio (ER).

CAF in receptor solution was plotted as the cumulative amount of CAF permeated through the skin versus time for all applied formulations. In the IVPT study (Section 2.5), the amount of CAF in the skin tissues was calculated from the HPLC analysis of extract solutions, giving the amount of CAF retained in the SC (tape strips 2–10) and the amount of CAF present in the skin tissue (epidermis, dermis).

Mass balance of IVPT experiments was determined by comparing the initial applied amount of CAF with the combination of CAF in the wash, tape strips 1–2 and 3–10 samples, the remaining skin tissue extracts, and the receptor compartment.

### 2.7. Anti-Cellulite Clinical Study

#### 2.7.1. Study Design

This study was a randomised, double-blind, placebo-controlled trial. Participants applied active and placebo formulations to their posterior thighs, one formulation per thigh, twice daily based on random allocation (computer-generated randomisation). We randomised the formulation of creams, either active or placebo, to assign left or right thighs to individual participants as detailed in the Products Section (Section 2.7.3). The study employed a repeated-measures design to evaluate the intervention over 12 weeks.

#### 2.7.2. Participant Recruitment Resources

A total of 24 participants (aged 18–55, BMI > 22) were recruited through advertisements on campus noticeboards and online advertising via the Curtin Medical School social media. We referred to previous studies [24,44], in which power was calculated at 84% for 42 legs (21 participants) with an effect size (E.S.) of 0.33 between 2 treatments (6 measurements) over 6 time points. Therefore, in our study, we found that an E.S. of 0.33 was detected with 24 participants (48 legs) at 80% power, using repeated measures of 2 treatments (active vs. placebo) over 4 time points (G*Power 3.1.9.4). Participants were excluded if they had used an anti-cellulite treatment in the last 3 months, had undergone major surgery, including liposuction, in the past year, or had scars on their posterior thigh. The study was approved by the Curtin University Human Research Ethics Committee (HSE2019-0521) and registered on the Australia and New Zealand Clinical Trials Registry (ACTRN12619001547134).

#### 2.7.3. Products

A batch of active and placebo was prepared and packaged into jars. Active was the optimised 2% CAF nano-cream formulation containing LAN (as determined from the IVPT study), whilst placebo was a nano-cream formulation containing only LAN. Participants received two jars of cream (one active and one placebo) and spatulas for each 4-week application period. The cream jars were labelled “A” and “B” by a study team member who prepared the randomisation schedule and was not involved in the data collection process. Participants received individual instructions to apply creams “A” and “B” to the designated legs. All cream ingredients were pharmaceutical grade and categorised as GRAS (Generally Regarded as Safe) by the U.S. FDA.

#### 2.7.4. Protocols and Clinical Assessment

On day 0 (baseline) and at 4, 8, and 12 weeks, the following set of standardised testing procedures, including photography sessions and measurements, was performed:

*Cellulite Appearance Evaluation*

To assess the effectiveness of the CAF cream, a validated tool for evaluating cellulite on the posterior thigh in clinical and experimental settings was established using a simple iterative process [45], based on a set of standardised photographs established in previous studies [45]. Images were coded and two evaluators and an independent moderator scored cellulite severity using a grading scale chart [46] to determine severity scores for each testing session. A standardised protocol was established by our group [45] and evaluators followed the steps outlined in this protocol, scoring each participant’s cellulite on the posterior thigh. During each photo session, the participant’s position and lighting were standardised, ensuring they were consistently photographed from the same angle by the same investigator. Both temperature (22–25 °C) and relative humidity (RH 40–50%) were regulated and lighting was appropriate for photographic evaluations.

*Thigh Circumference (T.C.) Measurement*

The investigator measured the thigh circumference three times (triplicate) at each follow-up time point using a standard measuring tape at a marked location 150 mm above the patella, the treatment site mid-point.

*Skin-fold Thickness (S.T.) Measurement*

Skin-fold thickness was measured at the treatment site mid-point using a plicometer (Harpenden body fat calliper; Baty International, Sussex, UK). Measurements involved gripping the skin with the thumb and index finger 1 cm from the mid-point, with the same investigator taking all measurements to ensure consistency.

*Participant Self-Assessment Questionnaire*

At weeks 4, 8, and 12, participants completed a questionnaire assessing their perception of the products and their effects on the skin. Participants answered a questionnaire using a five-point Likert scale, evaluating the products based on colour, odour, absorption, spreadability, stickiness, and skin sensation. They also answered a five-point Likert scale questionnaire on changes in dimpled appearance, level of moisture, elasticity, and skin smoothness.

At the end of week 12, participants also completed a seven-point global rating of change (GROC) scale, which assessed their perception of cellulite changes on their thighs based on the following options: “much worse, worse, somewhat worse, the same, somewhat better, better and much better” than prior to application of the creams.

*Procedure*

Participants who fulfilled the pre-screening inclusion criteria were invited to participate by informed consent procedures and instructed to maintain their regular routine (diet, exercise, tea, and coffee) throughout the 12-week study duration. Each participant was given two jars and spatulas, labelled “A” and “B” and educated on the location and volume of cream to apply twice daily (morning and night) to the posterior thighs. After application, each cream was to be rubbed into the skin for 30 s until fully absorbed.

After initial measurements (height, weight, thigh circumference, and skin-fold thickness) and photos for cellulite evaluation were taken, a schedule was set for a 4-week measurement and photo session. The study investigator sent reminders to participants via mobile phone before each session. This procedure was repeated at 8 and 12 weeks. During each visit, participants were required to return their cream jars for weighing to assess usage. Additional jars of cream were given for the next treatment period. At each visit, participants were asked about any skin reactions they had experienced. If any occurred, the study coordinator advised discontinuing cream use and the participant was monitored for 2 days until the reaction resolved, after which time they resumed using the cream.

### 2.8. Statistical Analysis

#### 2.8.1. IVPT

All experiments were conducted in triplicate. Data are expressed as mean ± SD (non-biological) (physical characteristics-related measurements) and mean ± SEM (biological- related experiments). Analyses were conducted using GraphPad Prism™ 9 software and two-way ANOVA with Tukey post hoc test. Significance was considered at *p* < 0.05.

#### 2.8.2. Clinical Study

The primary outcome measure was the cellulite severity score. Secondary outcomes included thigh circumference and skin-fold thickness. Participants were included in the analysis if they applied the creams throughout the 12-week study and completed the photo sessions and assessments for each study period. Participants who withdrew or did not finish the trial were replaced, with their data excluded from analysis.

Continuous outcomes were measured as mean ± S.D. Linear mixed models analysed data, comparing the effects of placebo and CAF creams over time. Effect sizes (Cohen’s d) compared CAF and placebo data at week 12 [47]. GROC was assessed using an independent *t*-test with Stata^®^ v16.0 (StataCorp, College Station, TX, USA) analyses.

## 3. Results

### 3.1. HPLC Assay Method Validation

The HPLC method provided a single CAF peak with good separation from any skin sample-related peaks. The retention time for CAF was 3.4 ± 0.1 min (of total was 6.0 min). The calibration curve for CAF was linear from 0.025 to 20 µg/mL (R2 = 0.9999). The LOD and LOQ for CAF determination, based on six sample and blank injections, were 6.5 ng/mL and 21.6 ng/mL. The acceptability criterion for precision repeatability was met with RSD < 5% [48] across high (20 µg/mL; 0.32%), medium (1 µg/mL; 0.42%), and low concentrations (0.025 µg/mL; 0.43%). The mass balance recovery of CAF was 97.51 ± 0.81%.

### 3.2. Nano-Cream Formulations Loaded CAF

All nano-cream formulations were successfully fabricated using hot emulsification of oil and water phases with homogenisation. CAF-loaded nano-creams demonstrated stable systems with suitable consistency for topical use. Their physical appearance was an opaque white pearl tone, homogeneous, smooth, washable, and non-greasy. They spread easily without separation during storage at room temperature. All nano-creams had a pH below 6, closely matching the pH of skin, which ranged from 5.55 to 5.98.

### 3.3. Physical Characteristics of CAF Nano-Cream Formulations

Visually, all nano-cream formulations had a gleaming and lustrous white pearl tone. In comparison, the marketed 2% CAF cream had an opaque ivory-goldish tone. At ambient temperature, they were homogenous, smooth in texture, easily washable, and did not form a greasy film on the skin upon application. All creams spread easily on the skin. There was no phase separation in any nano-cream formulations when observed immediately after manufacturing or during long-term storage (4 months) at room temperature. The droplet size ranged from 297.40 ± 5.60 to 470.73 ± 48.29 nm (Table 2). The droplet size of LAN was slightly larger than that of TR5 (Table 2). The pH of all nano-cream formulations was under 6, bringing them close to the pH of skin. Whilst the aqueous phase pH was in the range of 5.55 to 5.98 (Table 2).

### 3.4. Incorporation of Various Chemical Penetration Enhancers (CPEs): Effect on Skin Delivery

CAF was found in all skin regions (SC and E + D + F) and permeated through the skin to the receptor fluid following topical administration of all nano-cream formulations, CAF-marketed formulation, or CAF in aqueous solution (control).

**Figure 1:**
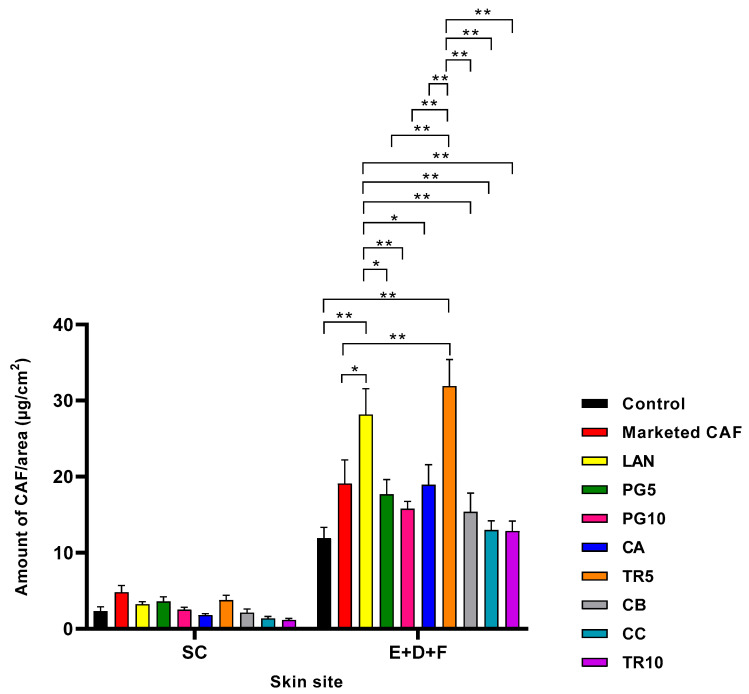
Skin penetration profile of nano-cream formulations compared to marketed topical product and CAF in aqueous control: the distribution of CAF in the SC and E + D + F (mean ± SEM; *n* = 8–9; * p < 0.01, ** p < 0.0001). SC = stratum corneum; E + D + F = epidermis, dermis, and follicles.

Figure 1 and Table 3 show the amount of CAF per area of each skin tissue at 8 h following administration of each CAF formulation. No statistically significant differences in CAF penetration into SC were observed among the applied formulations, marketed CAF topical product and the CAF control solution. However, significant differences were found in CAF levels penetrating to deeper skin regions (E + D + F) across formulations (28.18 ± 3.39; 19.10 ± 3.10; 11.90 ± 1.43 µg/cm^2^; * p < 0.01, ** p < 0.0001) (Table 3). Marketed CAF and CAF nano-cream formulations with TR5 and PG5 had the best CAF penetration in the SC (3.78 ± 0.64; 3.61 ± 0.61 µg/cm^2^, respectively), although the difference was not statistically significant (Figure 1 and Table 3). In the (E + D + F) region, the CAF distribution of LAN and TR5 nano-cream formulations were comparable (28.18 ± 3.39; 31.91 ± 3.49 µg/cm^2^, respectively; Table 3). Overall, there was a 2.3-fold increase in CAF in both SC and (E + D + F) regions for LAN compared to CAF in aqueous control.

**Figure 2:**
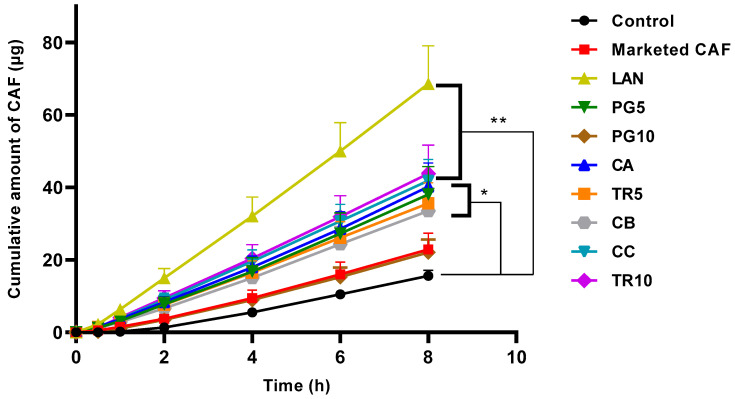
Cumulative amount of CAF penetrated in receptor solution over 8 h from all formulated topical creams (mean ± SEM; *n* = 8–9, * p < 0.05, ** p < 0.0001).

Figure 2 illustrates the permeation profile of CAF across formulated nano-creams, a marketed CAF product, and CAF in aqueous control. In the CAF nano-cream formulation, the inclusion of LAN demonstrated the highest CAF skin permeation (68.63 ± 11.59 µg, respectively; *p* < 0.0001), followed by TR10 and CC nano-cream formulations (43.80 ± 7.89; 41.87 ± 5.88 µg; *p* < 0.0001), respectively (Figure 2, Table 4). The cumulative amount CAF after 8 h (Figure 2) was significantly higher for the PG5, TR5, CA, and CB nano-cream formulations (38.08 ± 7.66; 35.63 ± 5.64; 40.20 ± 6.54, and 33.53 ± 6.35 µg, *p* < 0.001 respectively) compared to CAF in aqueous control (15.57 ± 1.57 µg) (Table 4).

There was no significant difference in CAF permeation through the skin over 8 h (Figure 2) for the PG10 nano-cream compared to CAF in aqueous control. However, a 1.28-fold increase in CAF was shown in the SC and epidermal–dermal–follicular regions from the PG10 formulation (Table 4).

The summary of experimental data on CAF skin permeation is presented in Table 4. LAN nano-cream formulation showed higher CAF flux and shorter lag time compared to the aqueous control and marketed CAF. Furthermore, LAN in this formulation improved CAF skin permeation by about 3.49-fold. The steady-state flux of CAF in the LAN nano-cream formulation was 8.829 ± 1.472 μg/cm^2^/h, compared to 2.533 ± 0.480 μg/cm^2^/h for the control CAF solution (Table 4). CAF from the LAN nano-cream formulation permeated 3.5 times faster than the control. The lag time was three times shorter for the LAN formulation than for the aqueous control. The ER for the PG5, CA, TR5, TR10, and CC formulations was approximately two, with their lag times being half that of the control CAF (Table 4).

Overall, nanoformulations in the form of cream manufactured with various CPEs significantly improved the cumulative amount of CAF delivered through the skin over 8 h. The LAN nano-cream formulation was considered for further investigation in the preclinical in vivo efficacy study. The addition of LAN has shown high flux and short lag time. Moreover, LAN formulation demonstrated the best capability in enhancing CAF permeation via the skin (3.49-fold) (Figure 2; Table 4).

### 3.5. Participants

Of the 29 female participants initially screened, 24 completed the study. Their ages ranged from 18 to 51 years (mean 31.68 ± 9.03) and the mean BMI was 29.04 ± 6.52 kg/m^2^. Seventeen participants identified as Asian or South Asian, six as European, and one as African. All 29 participants met the pre-screening requirements. Three withdrew due to study commitments, one due to a family reason, and another was excluded for having tattoos on her posterior thigh. During pre-screening, 87.5% had maintained a balanced diet consisting of meat and vegetables. Half of participants consumed 1 to 2 L of water daily. Coffee and tea were the most popular beverages, with most participants consuming more than one type.

### 3.6. Primary Outcome Measure

**Figure 3:**
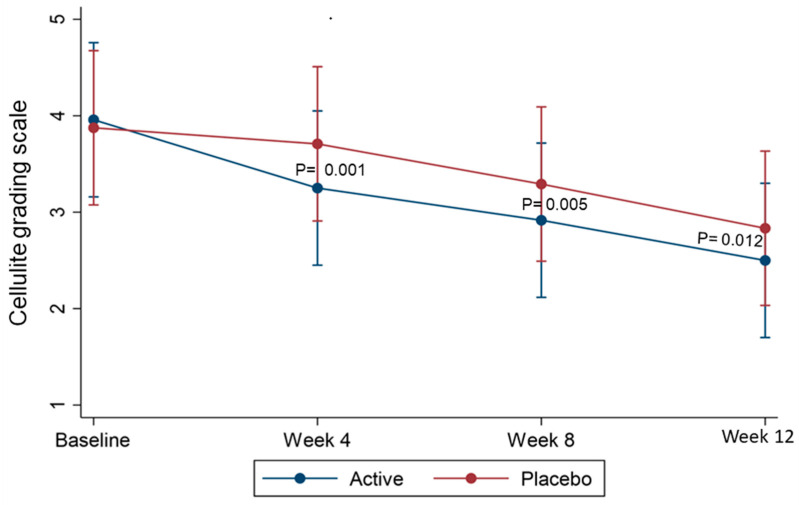
Mean (95% CI) cellulite severity grade from the female posterior thighs at baseline (week 0), with 12 weeks of treatment with active and placebo creams.

The primary outcome showed that the CAF cream significantly decreased the severity grade of cellulite compared to the placebo. After 12 weeks of twice-daily application, the CAF cream significantly reduced the severity grade of cellulite relative to the baseline (*p* < 0.05). Placebo treatment resulted in a limited reduction in cellulite (Table 5). A significant formula * time interaction occurred from week 4 to week 12 (Table 5, Figure 3). The Cohen *d* effect size at 12 weeks was 0.475, indicating a moderate effect with CAF cream versus placebo (Table 6).

### 3.7. Secondary Outcome Measures

Evaluation of T.C.

Table 5 summarises the mean circumference measurements for the CAF cream and placebo groups over time at each time point at the posterior thighs. After 12 weeks of using the CAF cream, participants showed a significant decrease in thigh size compared to the baseline (mean 56.01; 95%CI: 58.81–53.57; *p* = 0.023). The placebo group, however, also showed a notable reduction in thigh size (mean 55.70; 95%CI: 58.45–53.30; *p* = 0.031). No significant formula * time interaction effect was found at week 12 (*p* = 0.195).

Evaluation of S.T.

Table 5 reports the mean S.T. measures for the CAF cream and placebo cream at each measured time point. Starting from week 4, the anti-cellulite cream demonstrated a significant reduction in the S.T. of the posterior thighs in the CAF group compared to the placebo. That difference was maintained until the end of treatment (*p* < 0.001). After 12 weeks of twice-daily application, decreases were significant over baseline (mean 13.25; 95%CI: 11.99–14.52; *p* < 0.001) (Table 5), with an average reduction of −0.18 mm for posterior thighs (80% improved). The placebo group showed no significant baseline reduction. A significant formula * time interaction effect was noted between the groups (*p* < 0.001).

### 3.8. Participant Perception

Participants completed questionnaires regarding their satisfaction with the treatment and the nature of the products. Questionnaires were distributed and completed every 4 weeks.

*Treatment-related score*

Mean scores collected from self-evaluation questionnaires for the evaluation of hydration, elasticity, smoothness, and dimpled appearance for both creams are presented in Table 7.

After 4 weeks of using the CAF cream, participants indicated that their skin was better hydrated (mean 3.50; 95%CI: 3.17–3.83) than the baseline (*p* < 0.001). They also indicated that the CAF cream provided a smoother feeling on their skin (mean 3.71; 95%CI: 3.40–4.02) over the baseline (*p* < 0.001). Placebo treatment slightly improved skin hydration (mean 2.33; 95%CI: 2.00–2.66) and provided a less smooth surface on the skin (mean 2.83; 95%CI: 2.53–3.14). Both groups showed a significant formula*time interaction in improving skin hydration (*p* = 0.010; Table 7). Conversely, there was no significant difference between the two groups in the perception of improved skin texture (*p* = 0.809; Table 7).

At the end of the clinical trial, statistical analysis showed that the CAF cream (mean 3.83; 95% CI: 3.54–4.12) performed significantly better than the placebo (mean 3.08; 95%CI: 2.79–3.37) in terms of participants’ perceptions of skin elasticity. The participants expressed that “orange peel” dimpled appearance decreased on their posterior thighs following the CAF cream use (mean 3.96; 95%CI: 3.67–4.25). The comparison of the two groups showed a significant formula * time interaction effect for the elasticity measure and the dimpled appearance measure (*p* = 0.005; *p* < 0.001; Table 7).

*Product-related score*

Most participants agreed or strongly agreed that both creams had a good colour (*n* = 22), while half were neutral on whether the CAF cream (*n* = 11) and placebo (*n* = 12) had a pleasant smell. Fifty-four percent of participants agreed that the active cream was well absorbed. The same percentage said the CAF cream spreads quickly during application. Over ninety percent of participants were satisfied with the smoothing effect. The majority found the products to be non-sticky (*n* = 16).

### 3.9. Global Rating of Change (GROC)

At week 12, one participant reported no change in the appearance of cellulite on the posterior thigh after using the CAF cream, while four noted significant improvement after applying the placebo (Table 8). There was a significant difference in participants’ ratings of reduced cellulite appearance within 12 weeks of using a cream containing CAF (mean 6.08; 95%CI: 5.72–6.45) relative to placebo (*p* = 0.035).

### 3.10. Skin Irritation Reporting

No significant side effects occurred during the study. Most participants (79%, 19/24) experienced no skin reactions to either active or placebo creams. Two participants with CAF creams reported itching and prickling on their thighs, while three participants with placebo creams experienced the same symptoms.

## 4. Discussion

Nano-cream formulations typically consist of an aqueous phase, an oil phase, surfactants/co-surfactants (emulsifying agents), and active ingredients. Using nanotechnology to create semi-solid emulsions within a nano-sized range, which enhances skin penetration and improves stability and efficacy of the active ingredients compared to conventional formulations. The preparation of nano-cream formulations involves both high- and low-energy methods [50]. A nano-cream formulation is therefore likely to be more efficacious than existing cream products.

Our nano-cream formulations incorporating various penetration enhancers demonstrated enhanced CAF skin penetration and retention of CAF in all formulated nano-creams (Figure 1 and Figure 2). This study focused on formulas containing penetration enhancers such as fatty acids, esters, alcohols, and ether alcohols. These enhancers provide significant benefits due to their advantages in enhancing permeation capacity for passive skin transport [51,52,53,54], in combination with nano-sized globules to improve presentation to the skin surface. All nano-cream formulations were successfully fabricated using a low-energy method. Our CAF skin delivery compared favourably with previous studies utilising deformable liposomes [55,56,57] and microemulsions [58].

Figure 1 and Table 3 show that of the CPEs investigated, lanolin and Transcutol were significantly more effective than propylene glycol in delivering CAF to the skin from the nano-cream formulation. The LAN formulation also provided the highest cumulative permeation through the skin and caffeine flux over 8 h (Figure 2). LAN comprises cholesterol, ceramides, free fatty acids, and essential SC lipids [51,52,53]. It is a well-established component in topical pharmaceutical, personal care, and cosmetic products that have previously been shown to effectively enhance skin permeation. For example, Flockhart et al. [54] reported that LAN-derived nanoemulsions, which they termed lanosomes and consisted of 5 parts Laneth 20, 25 parts LAN, and 70 parts water, with globules sized 50–150 nm, were an effective topical delivery vehicle. Based on the IVPT study, the LAN nano-cream formulation was selected for evaluation of its anti-cellulite effect in the human clinical study.

The preclinical evaluation was conducted on the thigh area of suitable human participants, with a topical formulation administered and its effects evaluated over 12 weeks. One of the strengths of our study was the development and validation of a visual method for determining cellulite, which allows for an accurate assessment of changes over time due to treatment interventions. We involved two stages in our validation process [45]. The first stage was an open process without evaluator training. The second stage involved training and moderator review of grades. In the first stage, five evaluators assessed 24 photographs of right thighs using a cellulite severity chart to examine inter-rater reliability. In the second stage, paired evaluators with extra training examined the same photographs. Scores were independently moderated by a third person. The inter- and intra-rater reliability over 4 weeks were evaluated using intraclass correlation coefficients (ICCs). Having a reliable evaluation process is crucial for providing outcome measures that assess visual changes in clinical trials. Previous studies [8,46,59,60,61,62] evaluated the effectiveness of anti-cellulite topical creams containing CAF, using rating scores from validated visual scales [46,53,61,62,63,64,65,66]. However, inter-rater and intra-rater (test–retest) reliability when using a standardised grading system for scoring cellulite appearance changes was not reported [8,44,46,59,60,61]. To verify the results obtained, we generated an iterative process using a reliable and easy method to assess intervention effects during the clinical trial [45]. This process can be modified to evaluate different skin conditions using visual imaging. To the best of our knowledge, only Yoo et al. [62], who employed visual assessment grading scales from DERMAPRO SOP carried out reliability tests to validate the process. Roure et al. [67] used a similar approach to our experiment, beginning with formula development, followed by ex vivo and then in vivo efficacy testing of their CAF creams.

Compared to the baseline (week 0), the cellulite severity scores significantly decreased after 12 weeks for both the active and placebo group (*p* < 0.001; Table 5). However, there was a significant difference (*p* = 0.012) in cellulite severity scores between the active and placebo creams at 12 weeks, indicating the efficacy of caffeine in reducing visible signs of cellulite (Figure 3). This contrasts with Saman et al. [60] who reported that their anti-cellulite cream (1% caffeine, 5% L-carnitine, and 0.015% coenzyme A) provided the same effect as placebo after two months of application, with 90% of participants giving both creams a satisfaction score of 5.

Over the 12-week period, LAN nano-cream with CAF significantly reduced skin-fold thickness compared to the baseline (*p* < 0.001; Table 5), whilst the placebo group showed no statistically significant reduction. A significant treatment*time interaction (*p* < 0.001) favoured the active treatment. About 80% of participants experienced consistently reduced skin-fold thicknesses over the baseline with a small effect size (ES = 0.041).

By week 12, thigh circumference was significantly reduced for both active (mean −0.55) and placebo (mean −0.51) treatments, with no treatment*time interaction, indicating similar improvements for both groups. Reduction in thigh measurement is a key parameter for evaluating the effectiveness of anti-cellulite treatment. Several studies using CAF alone or with botanical extracts reported a significant decrease in T.C. compared to placebo groups [8,10,44,46,59]. Only Saman et al. [60] reported no significant T.C. reduction with the placebo.

Participants self-assessed the sensorial qualities and efficacy of the nano-cream via survey questionnaires, noting that both active and placebo creams significantly improved skin hydration, elasticity, smoothness, and reduced visible cellulite after 8 and 12 weeks (Table 7). There was a notable difference in perceived efficacy regarding improvements in skin moisture, elasticity, and smoothness after using the CAF cream compared to placebo. They also felt satisfied with reduced skin dimpling at the end of treatment (Table 7).

The perceived differences in these skin parameters between the two groups at weeks 8 and 12 were minor, with both creams showing similar results. Given that both creams had identical vehicle ingredients that are contributing to skin hydration and skin-smoothing effects, it was expected that both creams would be perceived favourably in these measures. This effect has been shown previously, for example, Byun et al. [8] reported that participants using their cellulite cream containing 3.5% CAF and xanthenes, had comparable increase in skin firmness between active and placebo groups, that they attributed to the massage effect from cream application [46,68]. Most reported that both creams had similar textures, which were indistinguishable when applied to the skin.

At the end of the study, participants assessed GROC, rating the anti-cellulite effect. They gave a higher score, 6.08 (95%CI: 5.72–6.45) to the active cream compared to placebo of 5.50 (95%CI: 5.14–5.86) resulting in a statistically significant difference between active and placebo cream (*p* < 0.001) for the overall impression of the anti-cellulite effect. Two participants experienced minor side effects from the anti-cellulite cream and three from placebo. Prickling and itching sensations were typical side effects of topical products, but there were no serious adverse events that caused treatment discontinuation.

Cellulite is a complex condition that tends to improve slowly [46]. No treatment is fully successful [69]. Several topical creams claiming anti-cellulite benefits are available, mainly from the methylxanthine family. Our study indicated that an initial assessment of our new CAF cream in a clinical setting suggests it may be a promising treatment for cellulite.

Our study had limitations, including participants’ compliance with applying creams daily and correctly and weight fluctuations that affected thigh measurements. Another contributory factor to error is that daily weight fluctuation led to a perceived variability in thigh measurements. However, the reduction in T.C. was not due to weight loss, as no overall weight reduction occurred.

In conclusion, we have successfully developed nanoemulsion-based semi-solid systems for skin delivery of CAF. The greatest flux was observed with the addition of LAN to the CAF nano-cream formulation, indicating the best capability to enhance CAF permeation through the skin. Our preliminary study showed that our novel anti-cellulite cream was safe, effective, and well-tolerated, both in objective and subjective assessments, when used continuously for 12 weeks. We demonstrated that our novel topical cream containing CAF effectively reduced the visible appearance of cellulite and skin-fold thickness (Table 5). Participants indicated an improvement in skin moisture, skin elasticity, and smoothness of the skin after using the CAF cream relative to placebo. The participants also felt satisfied with a diminished appearance of skin dimpling at the end of treatment (Table 7). Further research is now needed to gather additional clinical data to evaluate this treatment with a larger sample size, longer treatment duration, and longer post-treatment follow-up [25]. The treatment could also be evaluated at different body sites (buttocks and abdomen) and comparisons could be made with other existing treatments.

## Figures and Tables

**Table 1:** Composition and formula of CAF topical nano-cream formulations (all as % *w*/*w*).

INCI Name	Formula							
	LAN	PG5	PG10	TR5	TR10	CA	CB	CC
**Phase A**								
Cetearyl alcohol	3	3	3	3	3	3	3	3
IPM	1	1	1	1	1	1	1	1
Lanolin anhydrous	2	-	-	-	-	2	2	2
Dimethicone 350 CST	1	1	1	1	1	1	1	1
BHT	0.05	0.05	0.05	0.05	0.05	0.05	0.05	0.05
Extra virgin olive oil	3	3	3	3	3	3	3	3
Ceteareth-20	2	2	2	2	2	2	2	2
**Phase B**								
Propylene glycol	-	5	10	-	-	5	-	5
Carbopol^®^-940	0.15	0.15	0.15	0.15	0.15	0.15	0.15	0.15
EDTA	0.1	0.1	0.1	0.1	0.1	0.1	0.1	0.1
CAF	2	2	2	2	2	2	2	2
Transcutol^®^ P	-	-	-	5	10	-	5	5
**Others**								
Optiphen plus^®^	0.75	0.75	0.75	0.75	0.75	0.75	0.75	0.75
DL-alpha-tocopheryl acetate	0.5	0.5	0.5	0.5	0.5	0.5	0.5	0.5
TEA	qs	qs	qs	qs	qs	qs	qs	qs
Aqueous ad	100	100	100	100	100	100	100	100

INCI Name = International Nomenclature of Cosmetic Ingredient; ad = up to; qs = quantum satis (quantum sufficit/as much as is sufficient); CST = centistokes; CPE = chemical penetration enhancer. Cream base containing CAF with the addition of CPE: LAN = lanolin; PG5 = propylene glycol 5%; PG10 = propylene glycol 10%; TR5 = transcutol 5%; TR10 = transcutol 10%; CA = combination A-lanolin propylene glycol 5%; CB = combination B-lanolin transcutol 5%; CC = combination C-lanolin transcutol propylene glycol 5%.

**Table 2:** Physical characteristics of CAF creams (mean ± SD; *n* = 3).

Formula	Physicochemical Evaluation								
	Appearance	Colour	Consistency *	Homogeneity	Phase Separation	Texture	pH		Droplet Size (nm)
							Cream	Aqueous Phase	
**Marketed CAF**	Opaque	Ivory- goldish	Excellent	Homogenous	No	Smooth	6.84 ± 0.02	6.81 ± 0.01	>1000
**LAN**	Opaque	White pearl	Excellent	Homogenous	No	Smooth	5.64 ± 0.01	5.55 ± 0.01	425.00 ± 5.37
**PG5**	Opaque	White pearl	Excellent	Homogenous	No	Smooth	5.36 ± 0.02	5.57 ± 0.01	461.00 ± 4.91
**PG10**	Opaque	White pearl	Excellent	Homogenous	No	Smooth	5.59 ± 0.01	5.75 ± 0.01	470.73 ± 48.29
**TR5**	Opaque	White pearl	Excellent	Homogenous	No	Smooth	5.71 ± 0.02	5.65 ± 0.02	297.40 ± 5.60
**TR10**	Opaque	White pearl	Excellent	Homogenous	No	Smooth	5.85 ± 0.03	5.79 ± 0.01	380.10 ± 4.70
**CA**	Opaque	White pearl	Excellent	Homogenous	No	Smooth	5.66 ± 0.01	5.59 ± 0.01	331.60 ± 15.00
**CB**	Opaque	White pearl	Excellent	Homogenous	No	Smooth	5.61 ± 0.02	5.67 ± 0.01	332.03 ± 30.65
**CC**	Opaque	White pearl	Excellent	Homogenous	No	Smooth	5.91 ± 0.01	5.98 ± 0.01	350.73 ± 0.46

* excellent = satisfactory condition when the final product formulation meets the requirement and stabilises in firm texture as a topical semi-solid with a one-step production process [49].

**Table 3:** Skin distribution of CAF from nano-cream formulations, marketed product, and aqueous solution control (mean ± SEM; *n* = 8–9).

Formula	CAF Distribution in the Skin (µg/cm^2^)		ER
	SC	E + D + F	
Control: CAF in aqueous	2.34 ± 0.55	11.90 ± 1.43	1.00
Marketed CAF	4.81 ± 0.90	19.10 ± 3.10	1.68
LAN	3.23 ± 0.35	28.18 ± 3.39	2.30
PG5	3.61 ± 0.61	17.69 ± 1.94	1.49
PG10	2.53 ± 0.31	15.78 ± 0.95	1.28
TR5	3.78 ± 0.64	31.91 ± 3.49	2.51
TR10	1.17 ± 0.22	12.86 ± 1.30	0.98
CA	1.83 ± 0.18	18.95 ± 2.61	1.46
CB	2.15 ± 0.47	15.38 ± 2.45	1.23
CC	1.38 ± 0.25	12.98 ± 1.21	1.01

ER = enhancement ratio was a ratio of the mean total amount of CAF deposited in the skin (SC and E + D + F) from formulated creams/CAF in aqueous control.

**Table 4:** Summary of experimental data for CAF skin penetration/permeation parameters in nano-cream formulations (mean ± SEM; *n* = 8–9).

Formula	Cumulative Amount (µg)	Steady-State Flux (*Jss*; μg/cm^2^/h)	Permeability Coefficient (Kp; cm/h)	Lag Time (min)	ER
Control: CAF in aqueous	15.57 ± 1.57	2.533 ± 0.480	0.001 ± 0.001	53.22 ± 3.42	1.00
Marketed CAF	22.88 ± 4.50	2.827 ± 0.555	0.001 ± 0.001	44.88 ± 5.46	1.12
LAN	68.63 ± 11.59	8.829 ± 1.472	0.002 ± 0.001	21.78 ± 3.66	3.49
PG5	38.08 ± 7.66	4.960 ± 0.004	0.001 ± 0.001	28.14 ± 5.22	1.96
PG10	22.06 ± 3.55	2.920 ± 0.465	0.001 ± 0.001	45.84 ± 3.78	1.15
TR5	35.63 ± 5.64	4.556 ± 0.659	1.650 ± 0.292	34.50 ± 6.24	1.80
TR10	43.80 ± 7.89	5.191 ± 1.036	2.312 ± 0.565	23.22 ± 4.32	2.05
CA	40.20 ± 6.54	5.055 ± 0.870	0.001 ± 0.001	21.78 ± 3.06	1.99
CB	33.53 ± 6.35	5.539 ± 0.715	2.348 ± 0.521	16.50 ± 2.52	2.19
CC	41.87 ± 5.88	5.417 ± 0.748	2.146 ± 0.488	21.90 ± 5.46	2.14

ER = enhancement ratio was calculated based on the ratio of mean values of *Jss* of the formulations to CAF in an aqueous solution.

**Table 5:** Mean change in cellulite grading scale, thigh circumference, and skin-fold fat thickness at baseline, week 4, week 8, and week 12.

Measure	Week	CAF Cream	*p*-Value	Placebo Cream	*p*-Value	*p*-Value
		Mean (95%CI)	Baseline to Each Time Point	Mean (95%CI)	Baseline to Each Time Point	Cross-Sectional Between-Group Treatments Each Time Point
**Primary outcome measure**						
Cellulite grading scale	0	3.96 (3.16–4.76)	-	3.88 (3.08–4.67)	-	0.530
	4	3.25 (2.45–4.05)	<0.001	3.71 (2.91–4.51)	<0.001	0.001
	8	2.92 (2.12–3.72)	<0.001	3.29 (2.49–4.09)	<0.001	0.005
	12	2.50 (1.70–3.30)	<0.001	2.83 (2.03–3.63)	<0.001	0.012
**Secondary outcome measures**						
Thigh circumference (T.C.)	0	56.56 (59.45–54.05)	-	56.21 (59.05–53.74)	-	0.157
	4	56.09 (58.89–53.64)	0.050	55.96 (58.76–53.53)	0.295	0.613
	8	56.13 (58.96–53.68)	0.081	56.06 (58.86–53.61)	0.519	0.753
	12	56.01 (58.81–53.57)	0.023	55.70 (58.45–53.30)	0.031	0.195
Skin-fold thickness (S.T.)	0	13.43 (12.15–14.72)	-	13.47 (12.19–14.76)	-	0.418
	4	13.33 (12.05–14.60)	0.041	13.49 (12.20–14.78)	0.714	<0.001
	8	13.30 (12.03–14.57)	0.012	13.53 (12.24–14.82)	0.280	<0.001
	12	13.25 (11.99–14.52)	<0.001	13.54 (12.25–14.84)	0.168	<0.001

**Table 6:** Effect size (Cohen’s d) calculations for the CAF cream relative to the placebo cream.

Measure	Cohen’s d
Cellulite grading scale	0.475
Thigh circumference	0.068
Skin-fold fat thickness	0.041

**Table 7:** Average self-perception scores for hydration, elasticity, smoothness, and dimpled appearance at baseline, week 4, week 8, and week 12.

Measure	Week	CAF Cream	*p*-Value	Placebo Cream	*p*-Value	*p*-Value
		Mean (95%CI)	Baseline to Each Time Point	Mean (95%CI)	Baseline to Each Time Point	Cross-Sectional Between-Group Treatments Each Time Point
**Evaluation**						
**Hydration**	0	2.29 (1.96–2.62)	-	2.08 (1.75–2.41)	-	0.286
	4	3.50 (3.17–3.83)	<0.001	2.33 (2.00–2.66)	0.201	<0.001
	8	3.75 (3.42–4.08)	<0.001	3.25 (2.92–3.58)	<0.001	0.010
	12	4.08 (3.75–4.41)	<0.001	3.58 (3.25–3.91)	<0.001	0.010
**Elasticity**	0	2.50 (2.21–2.79)	-	2.42 (2.13–2.71)	-	0.621
	4	3.33 (3.04–3.62)	<0.001	2.67 (2.38–2.96)	0.138	<0.001
	8	3.67 (3.38–3.96)	<0.001	2.79 (2.50–3.08)	0.026	<0.001
	12	3.83 (3.54–4.12)	<0.001	3.08 (2.79–3.37)	<0.001	<0.001
**Smoothness**	0	2.50 (2.19–2.81)	-	2.38 (2.07–2.68)	-	0.468
	4	3.71 (3.40–4.02)	<0.001	2.83 (2.53–3.14)	0.008	<0.001
	8	3.88 (3.57–4.18)	<0.001	3.42 (3.11–3.72)	<0.001	0.008
	12	4.00 (3.69–4.31)	<0.001	3.96 (3.65–4.27)	<0.001	0.809
**Dimpled appearance**	0	1.58 (1.29–1.87)	-	1.63 (1.33–1.93)	-	0.801
	4	2.50 (2.21–2.79)	<0.001	2.00 (1.71–2.29)	0.023	0.002
	8	3.33 (3.04–3.62)	<0.001	2.71 (2.42–3.00)	<0.001	<0.001
	12	3.96 (3.67–4.25)	<0.001	3.17 (2.88–3.46)	<0.001	<0.001

Notes: Responses to the questionnaires on the CAF cream and placebo cream. Close-ended questions using 5-point Likert scale. A numerical value of 1 to 5, where 1 = strongly disagree; 2 = disagree; 3 = neither agree/nor disagree; 4 = agree; 5 = strongly agree.

**Table 8:** Global rating of change (GROC) following either CAF cream or placebo cream application.

	CAF Cream	Placebo Cream
**Much worse**	0	0
**Worse**	0	0
**Somewhat worse**	0	1
**About the same**	1	2
**Somewhat better**	4	9
**Better**	11	8
**Much better**	8	4

Notes: Close-ended questions using 7-point Likert scale GROC (“since applying the products, the cellulite on my thigh was: much worse, worse, somewhat worse, the same, somewhat better, better, much better than before application”).

## Data Availability

The original contributions presented in this study are included in the article. Further inquiries can be directed to the corresponding author.
